# Regulatory Adaptation of *Staphylococcus aureus* during Nasal Colonization of Humans

**DOI:** 10.1371/journal.pone.0010040

**Published:** 2010-04-06

**Authors:** Marc Burian, Christiane Wolz, Christiane Goerke

**Affiliations:** Interfakultäres Institut für Mikrobiologie und Infektionsmedizin, Universität Tübingen, Tübingen, Germany; Columbia University, United States of America

## Abstract

The nasopharynx is the main ecological niche of the human pathogen *Staphylococcus aureus*. Although colonization of the nares is asymptomatic, nasal carriage is a known risk factor for endogenous staphylococcal infection. We quantified *S. aureus* mRNA levels in nose swabs of persistent carriers to gain insight into the regulatory adaptation of the bacterium to the nasal environment. We could elucidate a general response of the pathogen to the surrounding milieu independent of the strain background or the human host. Colonizing bacteria preferentially express molecules necessary for tissue adherence or immune-evasion whereas toxins are down regulated. From the analysis of regulatory loci we found evidence for a predominate role of the essential two-component system WalKR of *S. aureus*. The results suggest that during persistent colonization the bacteria are metabolically active with a high cell surface turnover. The increased understanding of bacterial factors that maintain the colonization state can open new therapeutic options to control nasal carriage and subsequent infections.

## Introduction

*Staphylococcus aureus* is both a major human pathogen and a ubiquitous commensal and colonizer of the skin and mucous membranes. Although multiple body sites can be colonized, the anterior nares form the main ecological niche of this species. Approximately 20% of the healthy human population is persistently and 80% intermittently colonized with *S. aureus* in the nose [1]. Nasal carriage has been identified as a major risk factor for the development of subsequent mostly endogenous infections [2], [3].

Several bacterial factors were determined to be involved in *S. aureus* nasal colonization: the wall teichoic acid (WTA) [4], clumping factor B (*clf*B) [5], the capsular polysaccharide (*cap*) [6], the iron-regulated surface determinant IsdA (*isd*A) [7], and the autolysin SceD (*sce*D) [8]. All factors were so far studied *in vitro* or in animal models with the exception of the adhesin ClfB, whose causal involvement was shown in humans [5]. Expression of most virulence and adherence factors were shown *in vitro* to be directly or indirectly influenced by diverse regulators such as the accessory gene regulator (Agr), the alternative sigma factor B (SigB), and the *sae* locus [9], [10], [11], [12]. The composition of the cell envelope is modified by the regulatory action of *gra*RS (also known as *aps*XRS) [13], [14] or the essential regulatory system *wal*KR [15].

A better understanding of the bacterial factors maintaining the colonization state can be important for controlling nasal carriage and subsequent infections. In the present study transcript analysis using quantitative real-time PCR was performed directly on nose swabs from persistently colonized healthy individuals. We could show that in the human nose most global virulence regulators are not active, with exception of the essential two-component regulatory system WalKR. Furthermore, *S. aureus* nasal colonization is characterized by the expression of genes mediating adhesion, cell surface dynamics/remodeling, the expression of immune evasion genes and the lack of toxin transcription.

## Results

### Characteristics of nose specimens from healthy *S. aureus* nose carriers

**Figure 1:**
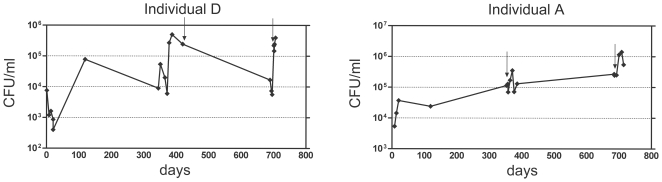
Bacterial loads in the noses of two persistent *S. aureus* carriers (individuals D and A) over time. Bacterial numbers are given in colony-forming units (CFU/ml) over a period of two years illustrated in days. Arrows indicate the time point of RNA isolation for quantitative real-time PCR.

For the analysis of *S. aureus* gene expression during human nose colonization we selected four persistently colonized individuals (A, B, C and D) with a history of repeated *S. aureus*-positive nose swabs. None of the volunteers received any antibiotics immediately before or between samplings. In general, *S. aureus* loads in the nose varied considerably over time, as exemplified for the two carriers A and D (Fig. 1). Specimens for transcript analysis were collected at two different time points: at the first sampling nose swabs were obtained from all four individuals, and at the second sampling 12 months later they were collected only from individual A and D. CFUs between 10^4^–10^5^/swab were detected in the noses of the volunteers at these time points. Bacteriological analysis revealed that all volunteers were colonized with a single *S. aureus* phenotype over time with the exception of individual A who carried a hemolytic and a non-hemolytic strain simultaneously at the second sampling (table 1). All carriers were colonized by distinct clones. Individuals A and D retained the same *S. aureus* strain over the whole investigation period. Detection of genes encoded on mobile elements revealed that none of the isolates contained a SCC*mec* cassette or the phage-encoded Panton-Valentine leukocidin gene. All nose isolates were lysogenic for *hlb*-converting phages encoding either staphylokinase (*sak*), the staphylococcal complement inhibitor (*scn*) and/or chemotaxis-inhibitor protein (*chp*) with exception of the ß-hemolysin (HLB) producing variant of individual A.

**Table 1:** Characteristics of nose isolates from four persistent carriers.

Individual	Sampling^a^	Phenotype^b^	GT^c^	*spa*-type	CC	Prophage content^d^	Accessory gene content^e^
A	1	non-hemolytic	2	t1239	CC30	Sa6, Sa3, Sa2	*sak*, *chp*, *scn*
A	2	non-hemolytic^f^	2	nd	CC30	Sa6, Sa3, Sa2	*sak*, *chp*, *scn*
A	2	hemolytic, ß	2	nd	CC30	Sa6, Sa2	-
B	1	hemolytic	241	t1200	excluded	Sa3	*sak*, *chp*, *scn*
C	1	hemolytic	253	t136	singleton	Sa5, Sa3	*sak*, *scn*
D	1	hemolytic	7	t015	CC45	Sa3	*sak*, *chp*, *scn*
D	2	hemolytic	7	nd	CC45	Sa3	*sak*, *chp*, *scn*

a. time point of sampling.

b. phenotype of *S. aureus* nose isolate on sheep blood agar plates.

c. genotype, determined by pulsed-field gel electrophoresis ß: positive for ß-hemolysin.

d. prophage content of isolates determined by identification of integrase gene type.

e. determined by standard PCR.

f. non-hemolytic phenotype 250 times more prominent, nd: not determined, - negative PCR result.

**Figure 2:**
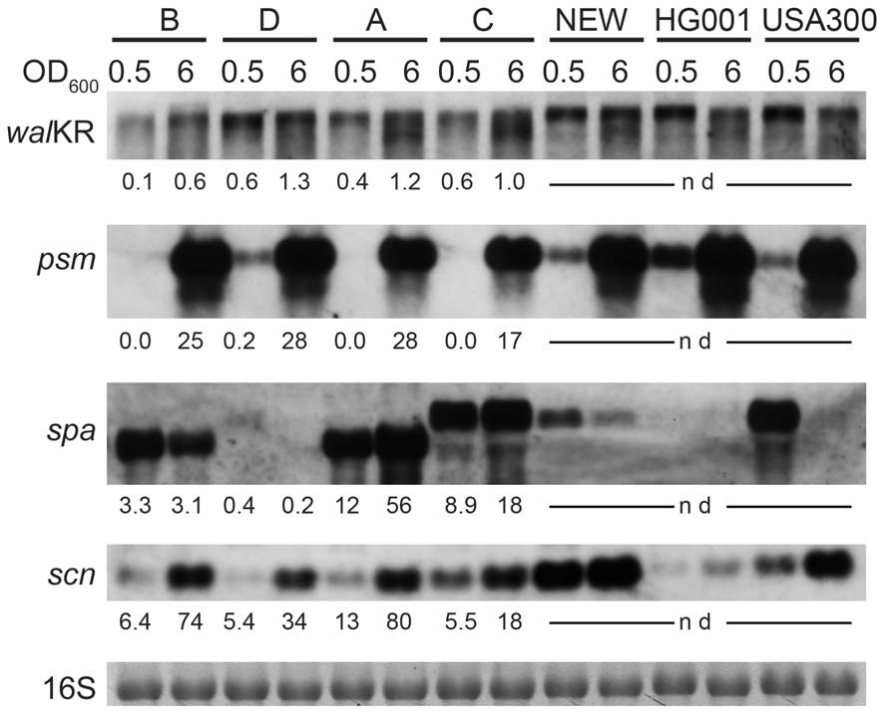
Expression pattern of the nose isolates of individuals A–D and of the laboratory strains Newman, HG001 and USA300 grown to the exponential (OD_600_ = 0.5) and post-exponential (OD_600_ = 6) growth phase. RNA was hybridized with probes specific for *wal*KR, *psm*, *spa* and *scn*. The 16S rRNA detected in the ethidium bromide-stained gels is indicated as a loading control in the lowest panel. The absolute transcript amounts determined by quantitative real-time RT-PCR are given below each lane. nd, not determined.

Most of the regulatory circuits controlling *S. aureus* gene expression have been described for prototypic strains. However, clinical isolates often exhibit a different regulation pattern. To elucidate the general properties of our four nose isolates we first analyzed the bacteria under *in vitro* conditions with quantitative RT-PCR (table S2) and for selected genes by Northern Blot analysis in comparison with the three characterized *S. aureus* strains Newman, HG001 and USA300 (Fig. 2). So far no expression data in *S. aureus* were available for the essential two-component system *wal*KR. We could show that it is maximally transcribed during the post-exponential phase in all nose isolates and in strain Newman. Next we determined transcription of phenol-soluble modulin (encoded by *psm*), which until now has only been described for caMRSAs such as USA300 [16]. We could detect an equally high expression in the post-exponential phase in all strains. In prototypic strains protein A is maximally expressed during the exponential phase due to the repressive action of the global regulator *agr* [17]. This could be confirmed but none of the four nose isolates showed the typical transcription pattern. Similar results were already obtained by us for CF isolates [18]. The immune-modulator molecule *scn* was reported to be transcribed mainly during exponential growth [19]. We could not reproduce this observation either in the nose isolates or in the three laboratory strains.

### Direct transcript analysis in nose swabs from *S. aureus* carriers

**Figure 3:**
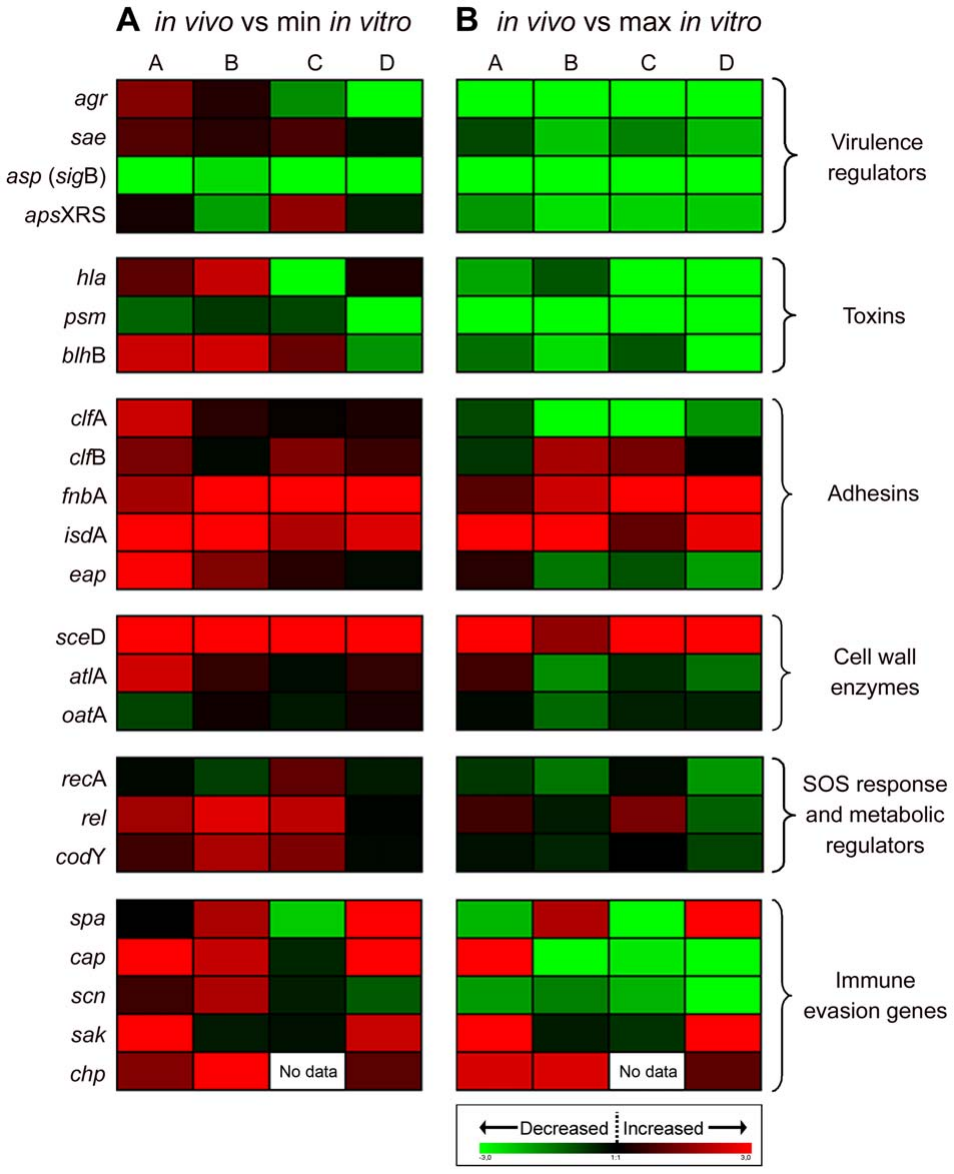
Direct transcript analysis of *S. aureus* genes in the nose of four persistent *S. aureus* carriers (A–D). Results are stated as the ratio of transcription *in vivo* versus minimal expression *in vitro* (A) and versus maximal expression *in vitro* (B). Changes in gene expression were normalized in reference to the constitutively expressed gene *gyr*B. Genes colored red are those which were up-regulated compared to in *vitro* and genes colored green are those which were down-regulated compared to *in vitro*. Black indicates the same expression levels *in vivo* and *in vitro*. Results are the means of two separate samplings. Gene name abbreviations see table S2. The color chart was generated using GENESIS software version 1.7.2. [51].

To profile the expression pattern of *S. aureus* during nasal colonization we performed quantitative transcript analysis directly on nose swabs obtained from persistent carriers. 30 different genes associated with a variety of cellular functions such as virulence regulation, toxin production, adhesion, cell wall dynamics and modification, immune modulation, SOS response and metabolic regulation were selected as targets (table S2). The transcript levels of all the target genes could be reliably quantified in these nose specimens with the exception of four genes: the mRNAs of *mpr*F, *dlt*A, *isa*A, and *ica* were below the detection level. In addition, these targets could only be detected from bacteria grown *in vitro* when 100-fold concentrated template RNA was analyzed, emphasizing the very low levels of these transcripts. The mRNA of the remaining 26 genes was quantified in the *ex vivo* material from the nose and compared to the transcription of the nose isolates during growth *in vitro*. In the first sampling two consecutive swabs from each of the four carriers were analyzed and the ratio of *in vivo* to minimal or maximal expression *in vitro* was calculated (Fig. 3A, B).

**Figure 4:**
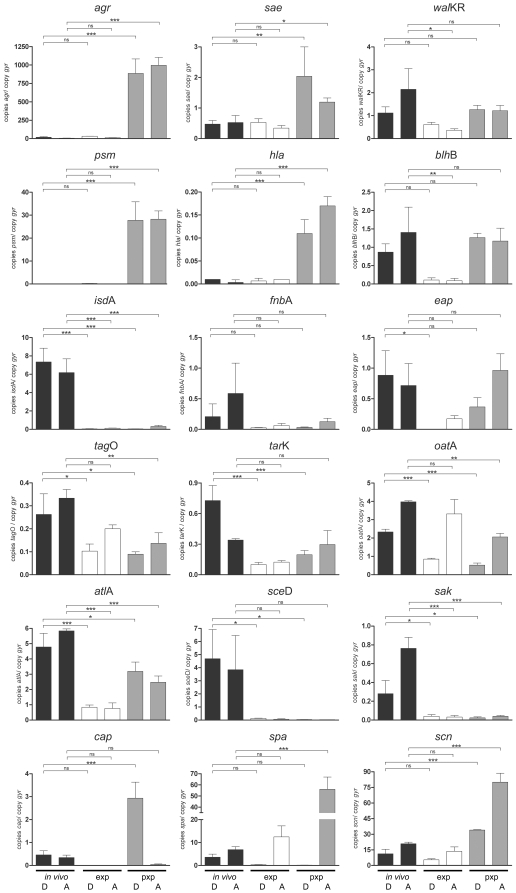
Direct transcript analysis of *S. aureus* genes in the nose of of two persistent carriers (individuals A and D) twelve months after the first sampling. Transcripts were quantified in reference to the transcription of *gyr*B directly in the nose swabs (*in vivo*, black columns) and after growth to the exponential phase (exp, white columns) and the post-exponential phase (pxp, gray columns). Values from three separate samplings (individual A) and four separate samplings (individual D), respectively were used to calculate the mean expression. Statistically significant differences between the *in vivo* and *in vitro* results are indicated: ns, not significant, P>0.05; * P<0.01 to <0.05; ** P<0.001 to 0.01; *** P<0.001. Gene name abbreviations see table S2.

**Figure 5:**
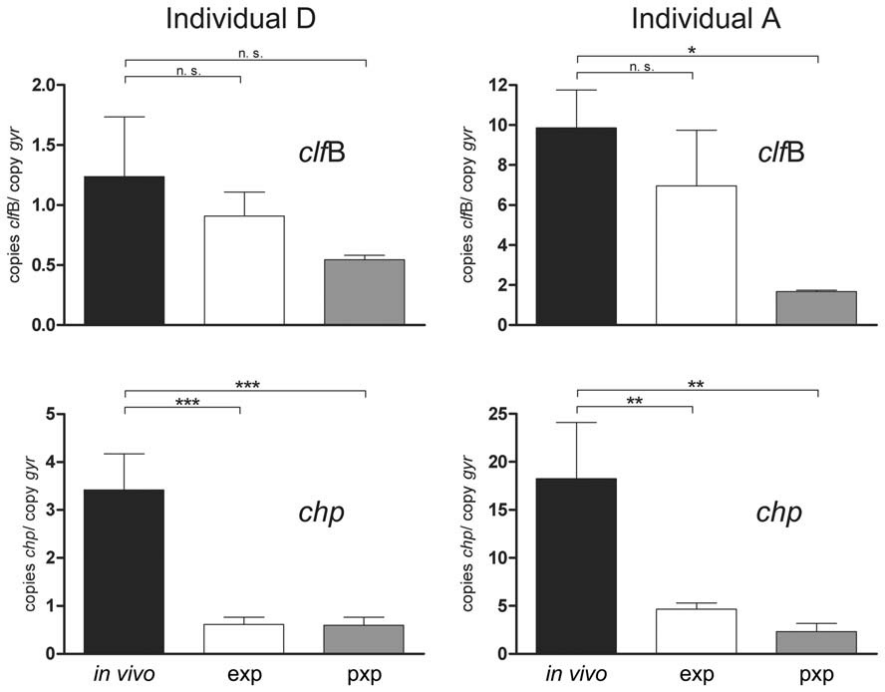
Direct transcript analysis of *S. aureus* clumping factor B (*clf*B) and chemotaxis inhibitory protein (*chp*) in the nose of two persistent carriers (individual A and D) twelve months after the first sampling. Transcripts were quantified in reference to the transcription of *gyr*B directly in the nose swabs (*in vivo*, black columns) and after growth in culture to the exponential phase (exp, white columns) and the post-exponential phase (pxp, gray columns). Values from three separate samplings (individual A) and four separate samplings (individual D), respectively were used to calculate the mean expression. Statistically significant differences between the *in vivo* and *in vitro* results are indicated: ns, not significant, P>0.05; * P<0.01 to <0.05; ** P<0.001 to 0.01; *** P<0.001.

Two of the four original carriers were sampled again 12 months after the first time point and multiple swabs were obtained for statistical analysis. The results are depicted as absolute copy numbers in reference to the housekeeping gene *gyr*B and in comparison with the expression profile *in vitro* (Fig. 4, 5). In the following the main results derived from both samplings are summarized according to the functional categorization of target genes:

### i.) Global regulators

Characteristically, *S. aureus* expresses adhesins in the early stages of growth, whereas toxins are produced in the late stages. A complex regulatory network is responsible for this differential gene expression, which is tightly controlled in response to cell density, energy availability and environmental signals. In our study we investigated the activity of five prominent regulators: *agr*, *sae*, *sig*B (detected as the tightly *sig*B-dependent *asp*), *gra*RS and *wal*KR. All regulators with the exception of *wal*KR were inactive during nose colonization, as shown by the low expression *in vivo* compared to maximal expression of the same strains *in vitro* (Fig. 3, 4). In contrast, the essential two-component system WalKR was highly transcribed in the noses of individual A and D similar to the maximal expression during the post-exponential phase, suggesting a role of this regulator during colonization (Fig. 4).

### ii.) Toxins

Invasion of host tissue, bacterial spread, and lysis of host cells is mediated by different types of *S. aureus* toxins. These molecules are usually expressed in high amounts during post-exponential growth (table S2). Transcript analysis of the alpha-hemolysin (*hla*), *psm* and a bi-component leukotoxin homologue termed *blh*B revealed that in all individuals these toxins were only poorly expressed *in vivo* (Fig. 3, 4).

### iii.) Adhesins

In *S. aureus*, cell-wall-anchored proteins and other adhesive molecules are implicated in binding to host matrix molecules and may therefore play an essential role in the establishment of colonization. This is consistent with the maximal expression of most adhesins during exponential growth (table S2). A pronounced transcription of the adhesins *clf*B, *fnb*A and *isd*A could be observed in the nose swabs of all investigated carriers (Fig. 3, 4). In contrast, transcript analysis of the post-exponentially expressed adhesins *clf*A and *eap* revealed a weak transcription during colonization.

The WTA of *S. aureus* is known to be an important colonization factor mediating adherence to epithelial and endothelial cells [20]. A multitude of enzymes are involved in WTA biosynthesis, but little is known about their regulation. We characterized the role of the WTA during colonization by analyzing the expression of *tag*O and *tar*K, which both contribute to WTA biosynthesis [4], [21]. We detected high transcriptional levels of both enzymes *in vivo* and a constant expression during growth *in vitro* (Fig. 4).

### iv.) Cell-wall modification enzymes

Enzymes involved in cell wall remodeling contribute especially to resistance against antimicrobial peptides. For instance, resistance to lysozyme is mediated by the O-acetyltransferase (*oat*A) [22] which was shown to be highly expressed during colonization (Fig. 3, 4). A pronounced *in vivo* expression was also detected for the autolysins *atl*A and *sce*D.

### v.) Stress response and metabolic regulators

Bacterial exposure to stress conditions triggers the SOS response in which the RecA protein is the key enzyme. To test whether exogenous pressure is exerted on *S. aureus* in the nose we measured *rec*A transcription. Expression was generally similar to or even below the minimal expression *in vitro* (Fig. 3), indicating the absence of SOS stimuli in the nasal environment.

Next we analyzed the activity of two metabolic regulators to gain insight into the physiological state of *S. aureus* during colonization. Bacteria adapt to amino acid insufficiency by a complex series of regulatory events known as the “stringent response” in which the bifunctional enzyme RelA is a key factor [23]. The repressor CodY has been described as inhibiting genes mainly involved in nitrogen metabolism [24]. Measurement of *rel*A *cod*Y transcription during colonization revealed a basal expression of both metabolic regulators in the human nose (Fig. 3).

### vi.) Immune evasion and immune modulatory factors

Protein A is a cell-wall-anchored protein involved in multiple immune-modulatory processes [25], [26]. In all nose specimens with the exception of individual C *spa* transcription could be detected suggesting a positive role of this molecule (Fig. 3, 4). *S. aureus* produces extracellular capsular polysaccharide, which is thought to protect the pathogen against opsonophagocytic killing by polymorphonuclear leukocytes [27]. Expression analysis of the *cap* operon revealed low-level production during nose colonization in most of the individuals.

In *S. aureus* prophages which integrate specifically into the ß-hemolysin (Hlb) gene are widely distributed, especially in nose isolates [28]. These prophages typically encode immune evasion molecules like SAK, SCIN, and CHIPS, which may contribute to the colonization capability of the bacterium by interacting with the host's innate immune system [29]. Transcript analysis of *sak* and *chp* revealed that these genes play an important role during persistent colonization since expression of both was clearly pronounced compared to the expression during growth *in vitro* (Fig. 3, 4, 5). In contrast, transcript analysis of the other phage-encoded immune-evasion gene *scn* revealed only a weak expression *in vivo*.

### Differences in *S. aureus* gene expression depending on host and time

*S. aureus* gene expression during colonization may be influenced by individual factors and therefore vary in the different human hosts. However, most of the analyzed transcripts were expressed in similar amounts in all carriers, suggesting a general response of the pathogen to the nasal environment. This was most obvious when analyzing transcripts which showed a highly variable expression during growth in culture, e.g., *spa* and *cap*. Individual A harbored a strain with high *in vitro spa* expression level (up to 70 copies spa/gyr) whereas the strain from individual D was a weak producer (0.4 copies spa/gyr). For both strains *in vivo* transcript levels of 4–8 copies spa/gyr could be detected. Similarly *cap* transcription leveled out during nose colonization (Fig. 3). *clf*B and *chp*, on the other hand, were not uniformly expressed in the noses of all individuals (Fig. 5).

Nasal colonization may necessitate a constant adaptation of the pathogen to its environment, which is probably reflected in a change in gene expression over time. However, when we reanalyzed *S. aureus* transcription in the noses of two of the original four carriers after 12 months, expression levels of most of the analyzed genes had remained unchanged in comparison to the first sampling. The only differently expressed genes were the *luk*-homologue *blh*B and the adhesin *eap*, which were more highly expressed at the second sampling (Fig. 4).

## Discussion

The human nose is the primary reservoir of *S. aureus*, and evolution of the species was probably driven by adaptation to this milieu. In general, bacteria react to environmental stimuli by differential gene expression which is controlled by various interacting regulatory networks. Our results indicate that in the nose important adhesive molecules (*clf*B, *isd*A, *fnb*A, *atl*A, *eap*, WTA), genes involved in cell surface dynamics/remodeling (*sce*D, *oat*A, *atl*A), and immune-modulatory factors (*sak*, *chp*, *spa*, *eap*) are prominently expressed, whereas major toxins (*hla*, *psm*) are not transcribed. Adherence to human desquamated nasal epithelial cells is an important factor in successful nasal colonization and was shown to be mediated by *clf*B, *isd*A and WTA *in vitro* [7], [30], in the nares of rodents in *in vivo* models [4], [31], [32] and in humans [5]. Thus, known colonization factors could indeed be proven to be activated on the transcriptional level in the nose by *S. aureus*. This supports our assumption that the results obtained from expression profiling can unravel essential factors for nose adaptation.

Nasal secretions form the first line of defence of the innate immune system against inhaled bacteria [33], [34]. The pathogen is able to counteract the most important mechanisms, namely antimicrobial molecules such as defensins and lysozyme, the complement system, immunoglobulins and phagocytes. *S. aureus* is lysozyme-resistant due to the combined action of OatA and WTA [22], both of which are produced by the pathogen during nose colonization. The highly transcribed staphylokinase is able to bind alpha-defensins, thus inhibiting their bactericidal effects [35]. In addition, SAK is able to activate surface-bound plasminogen into plasmin, which than has the ability to cleave the complement compound C3b and IgG, thereby efficiently preventing opsonization and subsequent phagocytosis by neutrophils [36]. Complement activation and opsonization are also blocked by the expression of protein A [25]. Surprisingly, we could not detect expression of the important complement inhibitor SCIN in the nose although it was shown that nasal secretions contain complement proteins [37].

To gain insight into the underlying regulatory network we analyzed five prominent regulators. The virulence regulators *agr*, *sig*B and *sae* were not activated during nose colonization. Accordingly, the typical target genes were only weakly expressed, toxins which are regulated by *agr* and *sae*, and *clf*A which is regulated by sigB [10], [38]. Surprisingly, the *gra*RS system which controls specific resistance mechanisms against antimicrobial peptides [13], [22] was not expressed either, despite various cationic antimicrobial peptides in the nasal fluid. This low *gra*RS expression is consistent with the high expression of *oat*A (generally down-regulated by *gra*RS) and the low expression of the two genes *dlt*A and *mpr*F (both strongly activated) [22]. However, another positively influenced factor, the autolysin *atl*A, was strongly expressed in the nose, indicating additional regulatory activators for this gene. Indeed, we could show that the essential, two-component system WalKR is active during nose colonization. Although only some of the target genes are known, it is interesting to note that *atl*A, *sce*D and *sak* are all positively regulated by walKR [39]. These factors were all expressed in the human nose. Thus, the WalKR regulatory system may play an important role in adaptation to the nasal environment. This is supported by our recent finding that the WalKR system is also the master regulator of adaptive gene expression in a cotton-rat model of nose colonization [40]. Furthermore, the high expression of cell wall metabolism genes (*atl*A, *sce*D) as well as the activity of WalKR strongly indicate that cell wall dynamics are critical for host-pathogen interaction and prolonged colonization of the human nose. However, little is known about the signaling cascade leading to activation of WalKR in *S. aureus*.

The physiological milieu of the human nose has an important influence on the adaptation of *S. aureus* to this ecological niche, and the host signals which are sensed by the pathogen are largely unknown. The expression of *isd*A is indicative of iron-limited conditions *in vivo*, since this molecule was shown to be highly iron-regulated [31]. The pathogen seems to be actively dividing in the nose environment, as suggested by the expression of many of the enzymes involved in cell-wall biosynthesis (*tag*O, *tar*K, *atl*A, *sce*D). This notion is additionally supported by the fact that most of the genes expressed in the human nose are those which are normally expressed *in vitro* during the exponential growth phase (*clf*B, *fnb*A, *isd*A, *sce*D, *chp*), indicating active *S. aureus* cells. On the other hand, we found little indication that the typical stress response factors (*rec*A, *rel*A, *cod*Y, *sig*B) are needed in the colonization process. They may be required in a more hostile environment, e.g. during infection.

In summary, we could elucidate here for the first time the expression pattern of *S. aureus* during asymptomatic colonization of the human nose. So far, direct transcript analysis has been performed during different kinds of infection [11], [18], [41], [42], [43], [44], [45]. Taken together the results indicate that *S. aureus* is able to specifically adapt to different niches in the human host. These findings significantly enhance our understanding of the complex host-pathogen interplay.

## Methods

### Ethics statement

The ethic committee of the University Hospital of Tübingen approved the study design. Written consent was obtained from all participants involved in the study according to their guidelines.

### Study population, bacteriological analysis, and characterization of bacterial isolates

Four healthy volunteers (two males and two females, median age 32 [range 26–43]) with repeatedly *S. aureus*-positive, high bacterial count nose swabs were selected for this study. Nose swabs were immediately used for RNA isolation without any subculturing of the bacteria. 10 µl of each swab were used for bacteriological analysis. Samples were quantitatively analyzed on sheep blood agar plates and *S. aureus* was identified by Staphaurex plus (Remel). All isolates were typed with pulsed-field gel electrophoresis [46] and *spa* typing as described [47]. Detection of PVL, mec, *sak* and the integrase type of prophages was done as described [28], [47], primers for *chp* and *scn* see Table S1.

### RNA isolation

For *in vitro* transcript analysis, nose isolates were grown overnight in CYPG (10 g/l casamino acid, 10g/l yeast extract, 5 g/l NaCl, 0.5% glucose and 0.06 M phosphoglycerate) [48], diluted to an initial OD_600_ of 0.05 in fresh medium and grown to the exponential (OD_600_ = 0.5) or post-exponential (OD_600_ = 0.5+4h) phase. Bacteria were harvested by centrifugation and dissolved in 1 ml Trizol reagent (Invitrogen). For *in vivo* transcript analysis, a cotton wool swab was moistened in 250 µl nuclease-free water and the left and right anterior nares of the human volunteers were swabbed. The swab was vigorously vortexed and 10 µl of the suspension were put aside for bacteriological analysis. The cotton wool was removed from the swab using sterile tweezers and both the suspension and the cotton wool were directly treated with 1 ml of Trizol LS reagent (Invitrogen).

Bacteria were lysed and RNA isolation was performed as described [41] with modifications to increase the amount of isolated RNA. First, precipitation was performed for 60 min at −20°C with the addition of 0.5 M ammonium acetate and 50 µg/ml GlycoBlue (Ambion). Second, after precipitation a prolonged centrifugation step of 1 h at 12,000 g was carried out. The dried RNA pellet was dissolved in 20 µl nuclease-free water.

To eliminate contaminating DNA each RNA sample was digested with 8 U of RNase-free DNaseI (Roche), 5 mM MgCl_2_, and 16 U of RNasin (Promega) for 30 min at 25°C. DNaseI treatment was stopped using DNase inactivation reagent (Ambion).

### Northern analysis

Northern blot analysis was performed as described [18]. As controls *S. aureus* strains Newman [49], HG001 formerly named RN1HG [24] and USA300 [50] were used.

### Quantitative RT-PCR

Sequence-specific RNA standards for quantification were prepared as described previously [41]. Briefly, gene-specific primers with a 5′-extension including the T7 promoter sequence (table S1) were used in a standard PCR. T7-driven *in vitro* transcription was performed in a standard transcription assay (T7-MEGAshortscript, Ambion). After subsequent DNase I treatment, RNA was recovered using the MEGAclear Kit (Ambion). RNA quantification was performed spectrophotometrically. RNA standards were diluted to 1×10^6^, 3.16×10^5^, 1×10^5^, 3.16×10^4^, 1×10^4^ and 3.16×10^3^ copies/µl. 1 µl of RNA standards, 1 µl (of a 1∶100 dilution) of *in vitro* and 3 µl of *in vivo* RNA were transcribed into cDNA using SuperScriptIII Reverse Transcriptase (Invitrogen) and 200 ng of random hexamer primers (Fermentas). cDNAs were frozen at −20°C using Eppendorf LoBind tubes (Eppendorf) for prolonged storage.

Quantitative real-time PCR was carried out using the LightCycler instrument and the LightCycler DNA amplification kit for hybridization probes or for SYBR Green (Roche) with cDNA diluted 1∶5 (*in vivo*) or 1∶10 (*in vitro*). Reaction mixtures were prepared using primers listed in table S1 or published primers [18], [41]. All primers were designed to account for possible gene polymorphisms (if necessary, wobbles were introduced, as in the case of *eap* and *blh*B) and were evaluated for optimal binding on the genome-sequenced *S. aureus* strains *in silico*. In the case of extensive polymorphisms of targets (e.g. *eap*) PCR efficiencies were additionally determined experimentally. Possible cross-reactions with *S. epidermidis*, a bacterial species often found alongside *S. aureus* in nose specimens, were excluded. The number of copies of each sample transcript was then determined with the aid of the LightCycler software. The specificity of the PCR reaction was verified on 3% agarose gels.

The absence of contaminating DNA was proven for each sample by quantitative real-time PCR using *gyr*B-specific primers. No amplification product could be detected in any of the samples.

### Statistical analysis

Statistical analysis was performed with the Prism 4.0 package (GraphPad Software) using the one-way ANOVA test and Bonferroni's multiple comparison post-test. P<0.05 was considered to be statistically significant.

## Supporting Information

Table S1 Oligonucleotide primers and LightCycler hybridization probes.
(0.16 MB DOC)

Table S2 S. aureus genes analyzed by quantitative real-time PCR.
(0.23 MB DOC)
