# Supplementary material for: Regulatory Adaptation of Staphylococcus aureus during Nasal Colonization of Humans
Source: PLoS One. 2010 Apr 6;5(4):e10040. doi: 10.1371/journal.pone.0010040 (PMC2850373; doi:10.1371/journal.pone.0010040)
Supplement: Table S1 — Oligonucleotide primers and LightCycler hybridization probes. (0.16 MB DOC) [file pone.0010040.s001.doc]

Table S1: Oligonucleotide primers and LightCycler hybridization probes

| Target gene | | GenBank acc.no. | | Primer | | Primer sequence | | Purpose |
| --- | --- | --- | --- | --- | --- | --- | --- | --- |
| RNAIII | X52543 | | agr-1189 | | CGATGTTGTTTACGATAGC | | qPCR | |
| (*agr*) |  | | agr-1436 | | CGACACAGTGAACAAATTC | | qPCR | |
| *aps*XRS | BA000018 | | T7-graR-907 | | TAATACGACTCACTATAGGGAGAATGATGATTCATGGCTTTG | | Standard construction | |
|  |  | | graR-1443 | | ATCGTATCGTCACCTTTT | | Standard construction | |
|  |  | | graR-1088 | | ATGTTGCTGGTATTGAAGAT | | qPCR | |
|  |  | | graR-1183 | | GCACCAATAAAACCCATC | | qPCR | |
| *wal*KR | CP000046 | | T7-yycG-1871 | | TAATACGACTCACTATAGGGAGACGTGAAAAGATTGAAGATGA | | Standard construction | |
|  |  | | yycG-2604 | | TCGGTGATTGGTTTGGTA | | Standard construction | |
|  |  | | yycG-2165 | | GAAAAGGGCTCCGTAAAT | | qPCR | |
|  |  | | yycG-2285 | | TCGTCGTCGCAATAATAAT | | qPCR | |
|  |  | | walKR-for | | GACGTGAAAAGATTGAAGATGA | | Northern probe | |
|  |  | | walKR-rev | | TCGGTGATTGGTTTGGTA | | Northern probe | |
| *blh*B | BA000018 | | T7-blhB-3647 | | TAATACGACTCACTATAGGGAGATTTCACTTCTCCACCATACT | | Standard construction | |
|  |  | | blhB-4282 | | TCAGCAGCAACGACTCAA | | Standard construction | |
|  |  | | blhB-4032 | | TTCTTGTCATANGTTGGATCATCG | | qPCR | |
|  |  | | blhB-4196 | | TGATAAGNCTCAACAAAAA | | qPCR | |
| *psm* | BA000033 | | T7-psm-2223 | | TAATACGACTCACTATAGGGAGAGCAACAAACTAACAAGACTC | | Standard construction | |
|  |  | | psm-2870 | | GTATTTCCCGTCCTTTTA | | Standard construction | |
|  |  | | psm2391 | | CATCGTTTTGTCCTCCTG | | qPCR | |
|  |  | | psm-2715 | | TCATCGCTGGCATCATTA | | qPCR | |
|  |  | | psm-for | | GAGCAACAAACTAACAAGACTC | | Northern probe | |
|  |  | | psm-rev | | GTATTTCCCGTCCTTTTA | | Northern probe | |
| *clf*B | AP009351 | | T7-clfB-98 | | TAATACGACTCACTATAGGGAGACCACATCAGTAATAGTAGGG | | Standard construction | |
|  |  | | clfB-652 | | CAGCAGCATTTACTACCG | | Standard construction | |
|  |  | | clfB-136 | | ATAGGCAATCATCAAGCA | | qPCR | |
|  |  | | clfB-270 | | TGTATCATTAGCCGTTGTAT | | qPCR | |
| *fnb*A | J04151 | | T7-fnbA-126 | | TAATACGACTCACTATAGGGAGACAATCTTAGGTACGGCAT | | Standard construction | |
|  |  | | fnbA-402 | | TGCTTCTTCAGTTGTTACT | | Standard construction | |
|  |  | | fnbA-160 | | GGAGCAGCATCAGTATTCTT | | qPCR | |
|  |  | | fnbA-308 | | AGTTGCAGTTGTTTGTGTTT | | qPCR | |
| *isd*A | BX571856 | | T7-isdA-2294 | | TAATACGACTCACTATAGGGAGATGAAAAGTAACAAAGATAATGA | | Standard construction | |
|  |  | | isdA-3135 | | CAAGCAACATCACAACCA | | Standard construction | |
|  |  | | isdA-2665 | | GCAGTTACAGCAGGTTTA | | qPCR | |
|  |  | | isdA-2782 | | CAGCAAAACCAAACAATG | | qPCR | |
| *eap* | AJ290973 | | T7-map-50 | | TAATACGACTCACTATAGGGAGAACAACAACTTTAGCATTAGG | | Standard construction | |
|  |  | | map-650 | | CGGTAATACCTCTATTTGATT | | Standard construction | |
|  |  | | map-98 | | AATAATAATGAAGCGTCTGC | | qPCR | |
|  |  | | map-402 | | CCTACTTTCAAATCGANAAC | | qPCR | |
| *sce*D | BA000018 | | T7-sceD-1226 | | TAATACGACTCACTATAGGGAGAGCACCTGATGTTGGATTT | | Standard construction | |
|  |  | | sceD-1704 | | GAATCGTAGCAGGAAATG | | Standard construction | |
|  |  | | sceD-1404 | | GTGGTGCTTCAACTTCTT | | qPCR | |
|  |  | | sceD-1497 | | CAACAGGTGCTAATGGAG | | qPCR | |
| *isa*A | BA000018 | | isaA-4487 | | TGATGAACCAGTAGCACC | | qPCR | |
|  |  | | isaA-4688 | | AATGGTCAAACTGCTGGT | | qPCR | |
| *atl*A | D17366 | | T7-atlA-1809 | | TAATACGACTCACTATAGGGAGATCTATTTGATTTGTCACGTC | | Standard construction | |
|  |  | | atlA-2897 | | ACCGACACCCCAAGATAA | | Standard construction | |
|  |  | | atlA-2024 | | CTTGTAGGTTCAGCAGTCA | | qPCR | |
|  |  | | atlA-2139 | | GCTTGTTCAGTAGTTGCTT | | qPCR | |
| *oat*A | BA000018 | | T7-oatA-1665 | | TAATACGACTCACTATAGGGAGATATCCTCTTTGCTATTCGC | | Standard construction | |
|  |  | | oatA-2431 | | CGCTATTTATTGTATCGTTG | | Standard construction | |
|  |  | | oatA-2224 | | CATTAGAACCGCAAAACC | | qPCR | |
|  |  | | oatA-2351 | | TGGGACAGATACACGACT | | qPCR | |
| *dlt*A | BA000018 | | dltA-1 | | TGGCGTTGAAAGACTAGGC | | qPCR | |
|  |  | | dltA-2 | | TTACGAACTCAGACTGGCG | | qPCR | |
| *mpr*F | AF145699 | | mprF-2602 | | ACGCTGAGTATTTAGGCT | | qPCR | |
|  |  | | mprF-2768 | | AGTCGCTCTAAATCCACG | | qPCR | |
| *tag*O | CP000046 | | T7-tagO-4642 | | TAATACGACTCACTATAGGGAGATGCCGCTGCCTTAGTAGT | | Standard construction | |
|  |  | | tagO-5256 | | GGTGGCGATACATACAAAAT | | Standard construction | |
|  |  | | tagO-4931 | | TTCCATCCTGCCAAAATA | | qPCR | |
|  |  | | tagO-5071 | | GAATGGAACTGCTAAGATAACA | | qPCR | |
| *tar*K | CP000046 | | T7-tarK-2722 | | TAATACGACTCACTATAGGGAGATCAAGAGAAATCAGGGGTC | | Standard construction | |
|  |  | | tarK-3412 | | CGGTGCGAATAGAATAACTTT | | Standard construction | |
|  |  | | tarK-2985 | | TTACGGATAGACGCAAATG | | qPCR | |
|  |  | | tarK-3105 | | TGTGATGGTCTAAAGCGAAC | | qPCR | |
| *ica*B | AF086783 | | T7-icaB-3985 | | TAATACGACTCACTATAGGGAGAACCGTGTAAGAAAAGCGA | | Standard construction | |
|  |  | | icaB-4534 | | TTCATCAAGCCATAAGGA | | Standard construction | |
|  |  | | icaB-4166 | | TCCAAAACGAAGTGTATG | | qPCR | |
|  |  | | icaB-4256 | | ATAATAAACCCAGTTGCC | | qPCR | |
| *rec*A | L25893 | | T7-recA-63 | | TAATACGACTCACTATAGGGAGAGGAGAAATCTTTCGGTAAAGGTGC | | Standard construction | |
|  |  | | recA-786 | | AGCTACTCTAAATGGTGGTGCCAC | | Standard construction | |
|  |  | | recA-F1 | | GCTCAAGCATTAGGCGTAGAT | | qPCR | |
|  |  | | recA-R1 | | CGTCCACCTGGTGTAGTCTCT | | qPCR | |
|  |  | | recA-FL | | TGGGAGACACTCACGTTGGTTTACA-F | | Hybridization probe | |
|  |  | | recA-LC | | Red640-GCTCGTTTAATGTCACAAGCGTTACG-ph | | Hybridization probe | |
| *rel*A | D76414 | | T7-rel-for | | TAATACGACTCACTATAGGGAGAGTATGATTGGTGTGGGAC | | Standard construction | |
|  |  | | relStandard-rev | | ACGATGTGCTAAAGGTGC | | Standard construction | |
|  |  | | rel-LC1-for | | GAAGCACATAAAGGTCAGT | | qPCR | |
|  |  | | relA-1566-rev | | TTGTTGTTCTTCTTTTGAGC | | qPCR | |
| *cod*Y | BA000018 | | T7-codY-for | | TAATACGACTCACTATAGGGAGACACCAAGTATGCCGAATG | | Standard construction | |
|  |  | | codY-LC1-rev | | TCTACTTCACTATGCTTCTCA | | Standard construction | |
|  |  | | codY-LC2-for | | AAAGGTATTGCGGTTGAT | | qPCR | |
|  |  | | codY-LC2-rev | | TTCTCCTCCACCTAAAAT | | qPCR | |
| *spa* | J01786 | | spa-254 | | TACTTATATCTGGTGGCGTAA | | qPCR | |
|  |  | | spa-561 | | GGTCGTCTTTAAGACTTTGA | | qPCR | |
| *cap* | U73374 | | T7-capA-59 | | TAATACGACTCACTATAGGGAGAATGGAAAGTACATTAGAA | | Standard construction | |
|  |  | | capA-710 | | TTGAACCCAATACAGGCAATCC | | Standard construction | |
|  |  | | cap-879 | | TTCCGAAGATTATGAGTTTGGA | | qPCR | |
|  |  | | cap-1006 | | AAGCGCGACAACTAATCCTAA | | qPCR | |
|  |  | | capFL | | TGCACCGATGAGATTCACTACAGTTTTTGG-F | | Hybridization probe | |
|  |  | | capLC | | Red640-GCGACTTTAACTGCTGTACCGTCTGCTTT-ph | | Hybridization probe | |
| *sak* | DQ530361 | | T7-sak | | TAATACGACTCACTATAGGGAGATAAGTTGAATCCAGGGTTTT | | Standard construction | |
|  |  | | sak-LClo | | CATCAAGTTCATTCGACAAAGGAAA | | Standard construction | |
|  |  | | sak-A | | TGTAGTCCCAGGTTTAATAGG | | qPCR | |
|  |  | | sak-F2 | | CGCGAGTTATTTTGAACC | | qPCR | |
|  |  | | sakFL | | CCATCAACTCCAGTCACATTTACCA-F | | Hybridization Probe | |
|  |  | | sakLC | | Red640-CAAATACGGGCCTGTTGGTTCA-ph | | Hybridization Probe | |
| *chp* | DQ530361 | | T7-chp-41410 | | TAATACGACTCACTATAGGGAGAGATGATTTAGACTCTCCTTT | | Standard construction | |
|  |  | | chp-41753 | | TAACGGCAGGAATCAGTA | | Standard construction | |
|  |  | | chp-41484 | | TTCAGCAAGTGGTGTATTC | | qPCR | |
|  |  | | chp-41701 | | ACCGTTTCCTACAAATGA | | qPCR | |
|  |  | | chp-for | | GGTTTGGCAAGTTATGAAATGTCTG | | PCR | |
|  |  | | chp-rev | | GTACACACCATCATTCAGCGAAAG | | PCR | |
| *scn* | DQ530361 | | T7-scn-42516 | | TAATACGACTCACTATAGGGAGAATACTTGCGGGAACTTTA | | Standard construction | |
|  |  | | scn-42830 | | TGCTTCGTCAATTTCGTT | | Standard construction | |
|  |  | | scn-42600 | | TTGCCAACATCGAATGAA | | qPCR | |
|  |  | | scn-42746 | | CATTGCTTTTTGACCTGAA | | qPCR | |
|  |  | | scn-for | | TAGATAAAAATGAGGCACAAGC | | PCR, Northern probe | |
|  |  | | scn-rev | | CATTGCTTTTTGACCTGAA | | PCR, Northern probe | |

F, fluorescein; Red640, LightCycler-Red 640-N-hydroxysuccinimide ester; ph, 3’phosphate

LightCycler hybridization probes were designed by TIB Molbiol, Berlin, Germany.
