# Supplementary material for: Regulatory Adaptation of Staphylococcus aureus during Nasal Colonization of Humans
Source: PLoS One. 2010 Apr 6;5(4):e10040. doi: 10.1371/journal.pone.0010040 (PMC2850373; doi:10.1371/journal.pone.0010040)
Supplement: Table S2 — S. aureus genes analyzed by quantitative real-time PCR. (0.23 MB DOC) [file pone.0010040.s002.doc]

Table S2. *S. aureus* genes analyzed by quantitative real-time PCR

| **Product** | **Gene** | **Function** | ***In vitro* timing nose isolates^a^** | ***In vitro* timing^b^** | **Regulation^c^** | **References** |
| --- | --- | --- | --- | --- | --- | --- |
| Virulence regulators |  |  |  |  |  |  |
| Accessory gene regulator | *agr* | Virulence regulator | pxp | pxp |  | [1] |
| *S. aureus* exoprotein-expression | *sae* | Virulence regulator | pxp | pxp |  | [2] |
| Alkaline shock protein | *asp* (*sig*B) | Strictly *sig*B regulated gene | pxp | pxp | *sig*B (+) | [3,4] |
| Antimicrobial peptide-sensing system | *aps*XRS | Virulence regulator | pxp | unknown |  | [5,6] |
| Regulator of cell wall metabolism and virulence | *wal*KR | Virulence regulator | pxp | unknown |  | [7] |
| Toxins |  |  |  |  |  |  |
| α-Hemolysin | *hla* | Pore forming hemolysin | pxp | pxp | *sig*B (-), *agr* (+), *sae* (+) | [3,8,9,10,11] |
| Bi-component leukotoxin homologue | *blh*B | Unknown | pxp | unknown | *sae* (+) | [10] |
| Phenol-soluble modulin | *psm* | Cytolytic peptides | pxp | pxp | *agr* (+) RNAIII-independent | [12] |
| Adhesins |  |  |  |  |  |  |
| Clumping factor A | *clf*A | Adhesion | pxp | pxp | *sig*B (+) | [3,9] |
| Clumping factor B | *clf*B | Adhesion | exp | exp | - | [13] |
| Fibronectin binding protein A | *fnb*A | Adhesion | constant | exp | *sig*B(+), *sae* (+), *agr* (-) | [10,14,15] |
| Iron-regulated surface determinant A | *isd*A | Adhesion | constant | unknown | *sig*B (-) | [3] |
| Extracellular adherence protein | *eap* | Adhesion/Immune modulation | pxp | pxp | *sae* (+), *agr* (+) | [10,16] |
| Cell wall and related enzymes |  |  |  |  |  |  |
| *Staphylococcus carnosus* exoprotein D | *sce*D | Lytic transglycosylase | exp | exp | *sig*B (+), *agr* (+), *sae* (+), *wal*KR (+) | [17,18,19] |
| Immunodominant staphylococcal antigen | *isa*A | Lytic transglycosylase | exp | unknown | *wal*KR (+) | [18,20] |
| Major autolysin | *atl*A | Bifunctional peptidoglycan hydrolase | pxp | unknown | *wal*KR (+), *aps*XRS (+) | [19,21] |
| O-acetyltransferase A | *oat*A | O-acetylation of peptidoglycan | exp | unknown | *aps*XRS (-) | [21] |
| D-alanine-D-alanyl carrier protein ligase | *dlt*A | D-alanine modification of teichoic acids | varies | unknown | *aps*XRS (+) | [21] |
| Multiple peptide resistance factor | *mpr*F | Modification of membrane lipids with L-Lysine | pxp | unknown | *aps*XRS (+)* | [5] |
| Teichoic acid glycerol | *tag*O | WTA biosynthesis | constant | unknown | - |  |
| Teichoic acid ribitol | *tar*K | WTA biosynthesis | pxp | unknown | *agr* (-) | [22] |
| Intercellular adhesion protein B | *ica*B | Cell-cell adhesion/Biofilm formation | nd | exp | *aps*XRS (+) | [21] |
| SOS response and metabolic regulators |  |  |  |  |  |  |
| Recombinase protein A | *rec*A | Homologues recombination/ DNA-repair | pxp | unkown | - |  |
| Relaxed response A | *rel*A | GTP pyrophosphokinase (stringent response) | pxp | unkown | - |  |
| Control of dpp | *cod*Y | Transcription pleiotropic repressor CodY | pxp | exp | *aps*XRS (-) | [21] |
| Immune evasion genes |  |  |  |  |  |  |
| Immunoglobulin G binding protein A | *spa* | IgG binding/Immune evasion | varies | exp | *agr* (-) | [8] |
| Capsular polysaccharide serotype 5 and 8 | *cap*A | Anti-phagocytosis | pxp | pxp | *agr* (+), *sig*B (+)  *sae* (-) | [2,3,9] |
| Staphylokinase | *sak* | Immune modulation (phage encoded) | constant | pxp | *sig*B (-),*agr* (+), *wal*KR (+) | [3,20,23] |
| Chemotaxis inhibitory protein | *chp* | Immune modulation (phage encoded) | exp | exp | *sig*B (-), *sae* (+) | [10,11,23] |
| Staphylococcal complement inhibitor | *scn* | Immune modulation (phage encoded) | pxp | exp | *sig*B (-), *agr* (+), *sae* (+) | [23] |

a. time point of maximal expression *in vitro* of the characterized nose isolates, b. time point of maximal expression in prototypic strains known from literature, exp: exponential growth phase, pxp: post-exponential growth phase, nd: not determined, c. Regulators influencing target gene expression: (+) activation, (-) repression, - unknown regulation, * regulation is strain-dependent in *S. aureus.*

References

1. Novick RP (2003) Autoinduction and signal transduction in the regulation of staphylococcal virulence. Mol Microbiol 48: 1429-1449.

2. Steinhuber A, Goerke C, Bayer MG, Doring G, Wolz C (2003) Molecular architecture of the regulatory Locus *sae* of *Staphylococcus aureus* and its impact on expression of virulence factors. J Bacteriol 185: 6278-6286.

3. Bischoff M, Dunman P, Kormanec J, Macapagal D, Murphy E, et al. (2004) Microarray-based analysis of the *Staphylococcus aureus* σ^B^ regulon. J Bacteriol 186: 4085-4099.

4. Goerke C, Fluckiger U, Steinhuber A, Bisanzio V, Ulrich M, et al. (2005) Role of *Staphylococcus aureus* global regulators *sae* and sigmaB in virulence gene expression during device-related infection. Infect Immun 73: 3415-3421.

5. Li M, Cha DJ, Lai Y, Villaruz AE, Sturdevant DE, et al. (2007) The antimicrobial peptide-sensing system *aps* of *Staphylococcus aureus*. Mol Microbiol 66: 1136-1147.

6. Kraus D, Herbert S, Kristian SA, Khosravi A, Nizet V, et al. (2008) The GraRS regulatory system controls *Staphylococcus aureus* susceptibility to antimicrobial host defenses. BMC Microbiol 8: 85.

7. Dubrac S, Msadek T (2008) Tearing down the wall: peptidoglycan metabolism and the WalK/WalR (YycG/YycF) essential two-component system. Adv Exp Med Biol 631: 214-228.

8. Dunman PM, Murphy E, Haney S, Palacios D, Tucker-Kellogg G, et al. (2001) Transcription profiling-based identification of *Staphylococcus aureus* genes regulated by the *agr* and/or *sarA* loci. J Bacteriol 183: 7341-7353.

9. Cassat J, Dunman PM, Murphy E, Projan SJ, Beenken KE, et al. (2006) Transcriptional profiling of a *Staphylococcus aureus* clinical isolate and its isogenic *agr* and *sar*A mutants reveals global differences in comparison to the laboratory strain RN6390. Microbiology 152: 3075-3090.

10. Rogasch K, Ruhmling V, Pane-Farre J, Hoper D, Weinberg C, et al. (2006) Influence of the two-component system SaeRS on global gene expression in two different *Staphylococcus aureus* strains. J Bacteriol 188: 7742-7758.

11. Kuroda H, Kuroda M, Cui L, Hiramatsu K (2007) Subinhibitory concentrations of beta-lactam induce haemolytic activity in *Staphylococcus aureus* through the SaeRS two-component system. FEMS Microbiol Lett 268: 98-105.

12. Queck SY, Jameson-Lee M, Villaruz AE, Bach TH, Khan BA, et al. (2008) RNAIII-independent target gene control by the *agr* quorum-sensing system: insight into the evolution of virulence regulation in *Staphylococcus aureus*. Mol Cell 32: 150-158.

13. Ni Eidhin D, Perkins S, Francois P, Vaudaux P, Hook M, et al. (1998) Clumping factor B (ClfB), a new surface-located fibrinogen-binding adhesin of *Staphylococcus aureus*. Mol Microbiol 30: 245-257.

14. Entenza JM, Moreillon P, Senn MM, Kormanec J, Dunman PM, et al. (2005) Role of σ^B^ in the expression of *Staphylococcus aureus* cell wall adhesins ClfA and FnbA and contribution to infectivity in a rat model of experimental endocarditis. Infect Immun 73: 990-998.

15. Saravia-Otten P, Muller HP, Arvidson S (1997) Transcription of *Staphylococcus aureus* fibronectin binding protein genes is negatively regulated by *agr* and an *agr*-independent mechanism. J Bacteriol 179: 5259-5263.

16. Harraghy N, Kormanec J, Wolz C, Homerova D, Goerke C, et al. (2005) *sae* is essential for expression of the staphylococcal adhesins Eap and Emp. Microbiology 151: 1789-1800.

17. Ziebandt AK, Weber H, Rudolph J, Schmid R, Hoper D, et al. (2001) Extracellular proteins of *Staphylococcus aureus* and the role of SarA and σ^B^. Proteomics 1: 480-493.

18. Stapleton MR, Horsburgh MJ, Hayhurst EJ, Wright L, Jonsson IM, et al. (2007) Characterization of IsaA and SceD, two putative lytic transglycosylases of *Staphylococcus aureus*. J Bacteriol 189: 7316-7325.

19. Dubrac S, Boneca IG, Poupel O, Msadek T (2007) New insights into the WalK/WalR (YycG/YycF) essential signal transduction pathway reveal a major role in controlling cell wall metabolism and biofilm formation in *Staphylococcus aureus*. J Bacteriol 189: 8257-8269.

20. Dubrac S, Msadek T (2004) Identification of genes controlled by the essential YycG/YycF two-component system of *Staphylococcus aureus*. J Bacteriol 186: 1175-1181.

21. Herbert S, Bera A, Nerz C, Kraus D, Peschel A, et al. (2007) Molecular basis of resistance to muramidase and cationic antimicrobial peptide activity of lysozyme in staphylococci. PLoS Pathog 3: e102.

22. Meredith TC, Swoboda JG, Walker S (2008) Late-stage polyribitol phosphate wall teichoic acid biosynthesis in *Staphylococcus aureus*. J Bacteriol 190: 3046-3056.

23. Rooijakkers SH, Ruyken M, van Roon J, van Kessel KP, van Strijp JA, et al. (2006) Early expression of SCIN and CHIPS drives instant immune evasion by *Staphylococcus aureus*. Cell Microbiol 8: 1282-1293.
